# New Insights in Cow’s Milk and Allergy: Is the Gut Microbiota the Missing Link?

**DOI:** 10.3390/nu14081631

**Published:** 2022-04-14

**Authors:** Elvira Verduci, Gian Vincenzo Zuccotti, Diego G. Peroni

**Affiliations:** 1 Pediatric Department, “Vittore Buzzi” Children’s Hospital, 20154 Milan, Italy; elvira.verduci@unimi.it (E.V.); gianvincenzo.zuccotti@unimi.it (G.V.Z.); 2 Department of Health Sciences, University of Milan, 20142 Milan, Italy; 3 Department of Clinical and Experimental Medicine, Section of Pediatrics, University of Pisa, 56126 Pisa, Italy

The present Editorial derives from the Special Issue “New Insights into Cow’s Milk and Allergy” recently published in *Nutrients*. Currently, in clinical practice, the management of pediatric patients affected by Cow’s Milk Protein Allergy (CMPA) involving the use of extensively hydrolyzed formulas (eHF) or, amino acid formulas (AAFs), represents a model of food allergies in children [1]. For children with CMPA, ESPGHAN guidelines recommend cow’s milk protein-based eHF as a first-line recommendation. In addition, although with different indications, some guidelines suggest rice eHF as an alternative in clinical practice for the treatment of infants with CMPA. In the Diagnosis and Rationale for Action against Cow’s Milk Allergy (DRACMA) guidelines, rice-based eHF formulas are found to be effective alternatives to cow’s milk protein-based eHF, when available. On the other hand, the ESPGHAN guidelines suggest using rice-based eHF in children who have no tolerance for casein- or whey-based eHF or for families adopting a vegan diet. Regarding soy-based formulas, there are few current studies conducted on a large number of children with non-IgE mediated allergies, while they are recommended in IgE mediated forms. Once tolerance to soy protein has been established and the child is >6 months old, a soy protein-based formula can be considered, as recommended by the guidelines.

However, a proportion of children allergic to cow’s milk proteins do not tolerate eHF and consequently are given the only hypoallergenic alternative, AAF. AAFs are also used in case of multiple food allergies associated with slow growth, severe forms of allergy such as FPIES, anaphylaxis or in case of severe symptoms at the onset of CMPA [2]. The ideal characteristics for an optimal hydrolyzed formula are an affordable cost, a correct protein (especially amino acids) and lipid content, no cross-reactivity and good palatability. In order to choose the right formula, aspects regarding both the child (age, clinical symptoms, CMPA endotypes) and the hydrolysate (palatability, cost-effectiveness ratio and availability) should always be considered. Hydrolysates also have a proactive action on modulating the immune system, improving the epithelial barrier function by strengthening it through small peptides derived from HF, which are able to increase the expression of the gene for tight junction proteins. This results in a reduction in the absorption of the antigen and its contact with intestinal immune cells, which in turn lower allergic symptoms. This mechanism is reinforced by nutraceutical supplementation that presents immune-modulating properties [3] and even though it is still widely discussed how and where children with this allergy can be vaccinated, normally the clinical situation allows to proceed with vaccinations [4].

It is well established that hypoallergenic formulas (eHF) have an important role in allergy and immune development, due to their impacts on gut microbiota. *Lactobacillus* and *Bifidobacterium* species are mostly found in fecal microbiota of healthy breastfed infants, with fewer other organisms such as *Bacteroides*, *Clostridium*, and *Enterobacteriacea*. CMPA is associated with gut dysbiosis and the development of other allergic conditions in later childhood. The hypothesis of causality, yet to be assessed, is that an early gut dysbiosis may disrupt regulatory mechanisms of the immune response, triggering pro-allergic processes and increasing the risk of allergy. In the pursuit of accurate and personalized medicine, modification of the gut microbiome deserves investigation as a potential strategy in CMPA management. Recently, eHF containing prebiotics, probiotics and symbiotics has been investigated.

Probiotics are defined as “live microorganisms which when administered in adequate amounts confer a health benefit on the host”, while prebiotics are “a substrate that is selectively utilized by host microorganisms conferring a health benefit”, lastly symbiotics are “a mixture comprising live microorganisms and substrate(s) selectively utilized by host microorganisms that confers a health benefit on the host”. Specific strains of probiotics were added to HAF or given as an adjuvant treatment to the formula. Several studies have evaluated the effect of eHF supplemented with probiotics, such as *Lactobacillus Rhamnosus* GG or *Lactobacillus casei* CRL431 and *Bifidobacterium lactis* Bb12, in order to assess the clinical course of allergic manifestations and tolerance acquisition in allergic children. A systematic review showed that probiotic supplementation may be associated with earlier acquisition of tolerance to CMP in children with CMPA (limited low-quality evidence). Moreover, prebiotics formula seems to enhance levels of *Bifidobacterium* species in infants with CMPA.

A recent systematic review and meta-analysis [5] evaluated whether AAF containing symbiotics (AAF-Sym) (*Bifidobacterium breve* M16-V and prebiotics, including chicory-derived oligo-fructose and long chain-inulin) vs. AAF without pre or probiotics could have a beneficial effect on clinical outcomes, including clinical symptoms and allergenicity, rates of infections, hospitalization, medication usage and gut microbiota colonization in infants with CMA. Regarding clinical symptoms and allergenicity, data from all included studies showed that both AAF-Sym and AAF were hypoallergenic. Clinical symptoms were less in both groups, also after a double-blind, placebo-controlled food challenge (DBPCCFC). A lower cumulative average percentage of infections in infants fed with AAF-Sym vs. AAF alone was detected from pooled analysis (13.6% vs. 27.8%, respectively). Meta-analysis showed that the proportion of infants who had infections was significantly lower with AAF-Sym than with AAF (OR 0.35 (95% CI 0.19 to 0.67), *p* = 0.001. Regarding the usage of emollients, known as protectives and dermatological medications, data from pooled analysis found a reduction in infants fed with AAF-Sym (−69%). Regarding microbiota, a pooled analysis showed that the average percentage of Bifidobacterium species was higher in infants who received AAF-Sym (44.0%) than those who received AAF (12.2%), whilst *Eubacterium rectale* and *Clostridium coccoides* was significantly lower (11.7% vs. 29.5%, respectively). Overall CMPA infants have shown an inverse colonization pattern compared to healthy breastfed children, with a higher proportion of unfavorable gut microbiota species. However, this review demonstrated that approach treatments, like using AAF-Sym formulas, might lead to a colonization pattern that appears to be closer to that seen in healthy breastfed infants [5]. The effectiveness of AAF-Sym compared to AAF includes a half rate of infants experiencing allergic symptoms (−48%) and a 37% reduction in the rate of symptoms per person-year. HAF is estimated to account for up to 38% of the healthcare costs of managing CMPA in the first year after diagnosis so the findings may have a major impact, not only on infants and their families, but also on the healthcare system [6].

Concluding, we can state that despite the precise mechanism of the symbiotic effects remaining unclear, the improvement in clinical symptoms and allergenicity, rates of infections, hospitalization, medication usage and gut microbiota colonization by AAF-sym is proven. The use of specific formulas HAF with prebiotics, probiotics and symbiotics might be considered in managing CMPA children. However, the need for further research to optimize treatment protocols for infants with CMPA are required, with particular attention in regard to microbiota modulation.
